# Local Literature Bias in Genetic Epidemiology: An Empirical Evaluation of the Chinese Literature

**DOI:** 10.1371/journal.pmed.0020334

**Published:** 2005-11-22

**Authors:** Zhenglun Pan, Thomas A Trikalinos, Fotini K Kavvoura, Joseph Lau, John P.A Ioannidis

**Affiliations:** 1Department of Rheumatology, Shandong Provincial Hospital, Jinan 250021, Shandong, China; **2**Clinical and Molecular Epidemiology Unit, Department of Hygiene and Epidemiology, University of Ioannina School of Medicine, Ioannina, Greece,; **3**Institute for Clinical Research and Health Policy Studies, Department of Medicine, Tufts-New England Medical Center, Tufts University School of Medicine, Boston, Massachusetts, United States of America,; **4**Biomedical Research Institute, Foundation for Research and Technology–Hellas, Ioannina, Greece; U.S. Centers for Disease Control and PreventionUnited States of America

## Abstract

**Background:**

Postulated epidemiological associations are subject to several biases. We evaluated whether the Chinese literature on human genome epidemiology may offer insights on the operation of selective reporting and language biases.

**Methods and Findings:**

We targeted 13 gene-disease associations, each already assessed by meta-analyses, including at least 15 non-Chinese studies. We searched the Chinese Journal Full-Text Database for additional Chinese studies on the same topics. We identified 161 Chinese studies on 12 of these gene-disease associations; only 20 were PubMed-indexed (seven English full-text). Many studies (14–35 per topic) were available for six topics, covering diseases common in China. With one exception, the first Chinese study appeared with a time lag (2–21 y) after the first non-Chinese study on the topic. Chinese studies showed significantly more prominent genetic effects than non-Chinese studies, and 48% were statistically significant per se, despite their smaller sample size (median sample size 146 versus 268, *p* < 0.001). The largest genetic effects were often seen in PubMed-indexed Chinese studies (65% statistically significant per se). Non-Chinese studies of Asian-descent populations (27% significant per se) also tended to show somewhat more prominent genetic effects than studies of non-Asian descent (17% significant per se).

**Conclusion:**

Our data provide evidence for the interplay of selective reporting and language biases in human genome epidemiology. These biases may not be limited to the Chinese literature and point to the need for a global, transparent, comprehensive outlook in molecular population genetics and epidemiologic studies in general.

## Abstract 2

Using the chinese literature as an example, John Ioannidis and colleagues show that selective reporting and language biases occur frequently in human genome epidemiology

## Introduction

Research conducted in non-English-speaking countries may be published either in English-language journals that are usually indexed in major international bibliographic databases or in domestic journals, many of which are not indexed in international databases. There is some empirical evidence that the decision to publish in international versus domestic journals may be influenced by the nature of the results: Significant results may be published in international journals, while nonsignificant results appear in the local literature, resulting in language bias (the “tower of Babel” bias) [1,2]. The opposite phenomenon, a reverse tower of Babel bias, nevertheless has also been described [3] in which most of the locally produced and published literature is spuriously statistically significant. Moreover, other investigators have questioned whether the inclusion or not of non-English studies makes any meaningful difference in the overall picture of the evidence [4].

The available evidence on these biases stems from the literature of randomized controlled trials. However, there are other fields in which language biases may be particularly important to appreciate. Genetics poses some special challenges. There are millions of polymorphisms in the human genome, and an exponentially increasing number of studies are trying to associate genetic polymorphisms with the risk of common diseases or treatment outcomes [5]. The risk conferred by each one of these genetic markers is usually small [5], with odds ratios between 1.1 and 1.4. Therefore, selective publication of studies with different results may potentially invalidate the overall picture about genetic risk factors. Moreover, there is major debate on whether there are differences in the strength of the genetic effects across people of different “racial” descent [6–8]. Language-related biases would tend to affect predominantly literature that refers to populations of specific “racial” descent, thus affecting the larger debate on “racial” descent differences.

The Chinese literature is a prominent example of possible bias, because a plethora of domestic scientific journals are not cataloged in international databases. China accounts for one-fifth of the world population, and this research is of major importance not only for China, but also internationally. It has been estimated that overall, for each internationally indexed publication from China, there are 18 publications in local nonindexed journals [9]. The consequences of potential selective publication and language biases for human genome epidemiology research and for biomedical research in general are unknown. Here we aimed to evaluate the extent to which genetic association studies are published in local Chinese journals not indexed in PubMed. We tried to understand whether the results of the Chinese literature differs from the results of the non-Chinese literature and what the implications would be for the total evidence on postulated epidemiological associations and inherent biases.

## Methods

### Definitions

The primary comparison addressed the results of Chinese versus non-Chinese studies. “Chinese studies” refers to studies performed in the People's Republic of China, regardless of the language of publication. All of them have been performed in people of Chinese descent. Chinese studies are further classified according to whether they are indexed in PubMed or not. “Non-Chinese studies” refers to studies performed outside of China, regardless of the language of publication and regardless of the “racial” descent of the studied populations. Non-Chinese studies are further classified according to whether they evaluated people of Asian or non-Asian descent.

### Database of Meta-Analyses of Gene-Disease Associations

We used published meta-analyses of gene-disease associations with binary outcomes and unrelated subjects. Whenever a publication provided data on more than one “racial” descent group, these were split and counted as separate studies. We started from a dataset of 55 meta-analyses previously used in an evaluation of differences between small and larger genetic association studies with binary outcomes [10]. The exact search strategy and eligibility criteria for these meta-analyses have been described previously [10,11]. For each one of them, we updated searches until December 2004, in order to identify more recent meta-analyses on exactly the same topic and containing more studies. More comprehensive meta-analyses replaced the older ones. Then we focused only on meta-analyses in which at least 15 non-Chinese studies were already available. We took this approach because there is evidence that the early literature on gene-disease associations often provides unreliable, inflated results [11,12]. Moreover, Chinese studies may not appear for at least a few years after the appearance of the first non-Chinese studies, thus meta-analyses with few non-Chinese studies may not have had any Chinese studies published yet. Meta-analyses were selected regardless of whether or not they already included any studies from China or individuals of Chinese ethnic descent. None of these meta-analyses had access to Chinese journals not indexed in PubMed, and all included studies were PubMed-indexed.

### Search for Additional Studies from the Chinese Literature

For each of the eligible meta-analyses, we searched the national Chinese database of biomedical literature (last search December 2004) for potentially additional gene-disease association studies published in local Chinese journals that would fulfill the eligibility criteria of the original meta-analysis. The Chinese Journal Full-Text Database covers 8,000 journals since 1994, and it is accessible with username and password via the Web site of Tsinghua University. We excluded family-based studies, since they are based on linkage analyses, and these have also been excluded from the original meta-analyses as well.

The search strategy for each topic used the name of each genetic marker (using the abbreviated name of the gene, the expanded name of the gene, and the polymorphism) in combination with terms pertaining to the disease and/or outcome of interest. Retrieved abstracts and articles were further screened for eligibility by the same native Chinese investigator (ZP) who performed the literature search. When in doubt, two other investigators (JPAI and JL), one of them Chinese-speaking (JL), decided on the study's eligibility.

### Data Extraction

From each eligible Chinese study, we recorded the name of the first author, journal of publication, year of publication, ethnic descent, and data on the 2 × 2 table for the association (data necessary to derive the crude odds ratio and standard error thereof for the probed association). For consistency, the same genetic contrast was used as in the original meta-analysis. We also recorded whether the study was also indexed in PubMed.

We also examined, in each Chinese article, whether the disease was defined with specific criteria, whether any effort was described to ensure that the controls were indeed disease-free or otherwise appropriate, whether it was specified that genotyping was performed blinded to the clinical status, whether there was any mention that the disease-free controls were tested for conformity to Hardy-Weinberg equilibrium, whether any authors were involved from countries other than China, and whether the article was published in an international or national versus a local journal.

Data extraction was performed by a native Chinese investigator (ZP). Key data were independently verified by a second investigator (FKK) whenever tables in English were available or from another Chinese-speaking investigator (JL) otherwise. The few discrepancies were discussed and consensus was reached with a third arbitrator.

### Data Analysis

Descriptive statistics summarized the number of studies, total sample size, number and percentage of studies with statistically significant results on their own, and year of publication. Sample sizes were compared between groups of studies with the Mann-Whitney U test and with median regression adjusted for topic (bootstrap *p*-values). The proportion of studies with statistically significant results was compared with the χ^2^ test.

For each meta-analysis and for each group of studies, we estimated the summary odds ratio with inverse variance random effects models, which allow for between-study heterogeneity and incorporate it in the calculations [13]. We tested for between-study heterogeneity with the χ^2^-distributed *Q* statistic (considered significant at *p* < 0.10) [13], and estimated its extent with the *I* ^2^ statistic. *I* ^2^ ranges between 0% and 100% and represents the proportion of between-study variability that can be attributed to heterogeneity rather than chance (considered large for values of 75% and higher) [14]. Given the prominent differences in effect sizes between different groups of studies, it was considered inappropriate to obtain an overall summary effect including all of them. Instead, we estimated whether the results of different groups of studies differed between themselves beyond chance. A standardized *z*-score statistic was employed, as previously described [15].

For Chinese studies, for each study we estimated the probability that it would have a formally statistically significant result at the α = 0.05 level, conditional on the sample size of its case and control groups, the genetic marker frequency in the controls, and the summary genetic effect seen across Chinese studies. This calculation was performed as a regular power calculation for a case-control study. The sum of these probabilities across Chinese studies (the expected number of studies with statistically significant results) was then compared to the observed number of statistically significant findings using a χ^2^ test.

We also compared PubMed-indexed versus not PubMed-indexed Chinese studies as to all the other study and quality characteristics mentioned in the Data Extraction section above.

Analyses were conducted in Intercooled Stata 8.2 (Stata Corp., College Park, Texas, United States) using the *metan* module. *p*-Values are two-tailed.

## Results

### Data on Chinese and Non-Chinese Studies

Thirteen published meta-analyses were found with at least 15 non-Chinese studies [16–26]. Data on any Chinese studies could be retrieved for 12 of those, and these 12 topics are considered from now on (for the association of *DRD2* TaqIA polymorphism with alcoholism [26], no Chinese study was identified; Table 1). Overall, there were 161 eligible Chinese studies, only 20 of which were indexed in PubMed. Of the 20 Chinese studies indexed in PubMed (two on ID1, two on ID2, one on ID3, two on ID4, five on ID10, one on ID11, and seven on ID12; Table 1), only six had already been included in the published meta-analyses (one on ID11 and five on ID12), while the others were more recent; only seven of the 20 were published in full-text English journals. Of the 309 non-Chinese studies already included in the published meta-analyses, 44 pertained to populations of Asian descent (Japan, *n* = 25; Korea, *n* = 7; Chinese people outside of China, *n* = 5; Taiwan, *n* = 4; Malaysia, *n* = 2; and Singapore *n* = 1), and 265 to people of non-Asian descent (Figure 1).

**Figure 1:**
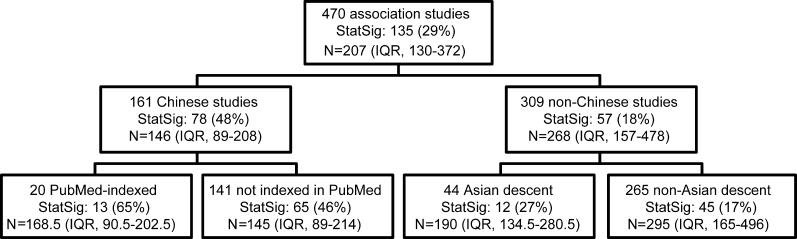
Categorization of the Examined Genetic Association Studies IQR, interquartile range; N, sample size (as median and interquartile range); StatSig, statistically significant at the 0.05 level.

**Table 1:**
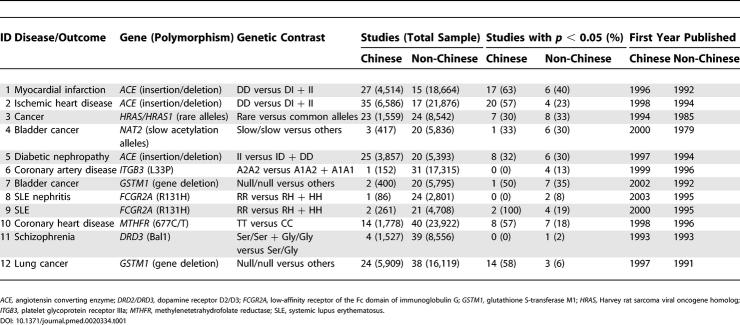
Eligible Meta-Analyses

For six topics we retrieved an extensive Chinese literature from the Chinese database (14–35 studies for each), while for the other topics the Chinese studies were sparse (four or fewer studies per topic) (Table 1). Chinese data were typically sparse if the disease was relatively uncommon in China compared with other countries, e.g., bladder cancer (bladder cancer is almost 10-fold less common in China than in Europe or the United States) [27] and alcoholism (at least until the early 1990s) [28]; or if the disease was not very common globally (e.g., systemic lupus erythematosus and schizophrenia). Chinese studies were plentiful if the disease was common (e.g., cancer in general, lung cancer, coronary heart disease, and diabetic nephropathy), with the exception of the postulated association of the *ITGB3* gene with coronary artery disease, for which only one Chinese study was available.

With one exception, where the first Chinese study was published in the same year as the first non-Chinese study, the first Chinese study always appeared with a considerable time lag compared with the remaining world literature (2–21 y; Table 1).

### Study Sample Sizes

The sample size for Chinese studies was significantly smaller than for non-Chinese studies (*p* < 0.001 both by U test and topic-adjusted median regression; Figure 1). Although non-Chinese studies of non-Asian descent populations overall seemed to be larger than studies on non-Chinese studies of Asian descent populations (*p* < 0.001 by U test), the difference was lost after adjusting for topic (*p* = 0.72). Chinese studies indexed or not indexed in PubMed did not differ in sample size (*p* = 0.79 by U test, *p* = 0.55 by median regression; Figure 1).

### Statistically Significant Results

Overall, 78 (48%) of the 161 Chinese studies had formally statistically significant results. There was some heterogeneity in this proportion across topics (exact *p* = 0.041). Conversely, only 57 (18%) of 309 non-Chinese studies had significant results, despite the larger sample size, and the percentage differed greatly across the 12 topics (exact *p* < 0.001). As shown in Figure 1, the proportion of formally statistically significant studies differed between PubMed-indexed Chinese studies, non-PubMed-indexed Chinese studies, non-Chinese studies of Asian-descent populations, and non-Asian studies (65%, 46%, 27%, and 17%, respectively; *p* < 0.001 by χ^2^). None of the five studies on Chinese-descent people living outside of China had statistically significant results.

### Changes in Study Sample Sizes and Significant Results over Time

The sample size of Chinese studies increased over time (Spearman correlation coefficient for publication year and sample size, 0.32, *p* < 0.001), while this was not seen for non-Chinese studies (correlation coefficient 0.00, *p* = 0.95). As for non-Chinese studies, the proportion of Chinese studies with formally significant results did not increase over time; if anything, there was a trend towards decrease (47/89 [53%] in 1993–2000 versus 41/72 [43%] in 2001–2004; *p* = 0.27).

### Comparison of Genetic Effects

Table 2 summarizes the genetic effect sizes. As shown, whenever there was a sizeable literature of Chinese studies, the gene-disease association was always formally significant in both non-Chinese and Chinese studies, but Chinese studies always showed a larger genetic effect than the non-Chinese studies (Figure 1). In five of the six topics the observed difference was even beyond chance (*p* < 0.05 on the *z*-score). Even with limited data, Chinese studies suggested larger estimates than non-Chinese studies also in the other three topics where there was some overall evidence for the presence of a gene-disease association; the genetic effect difference was beyond chance in one of the three topics (Table 2).

**Table 2:**
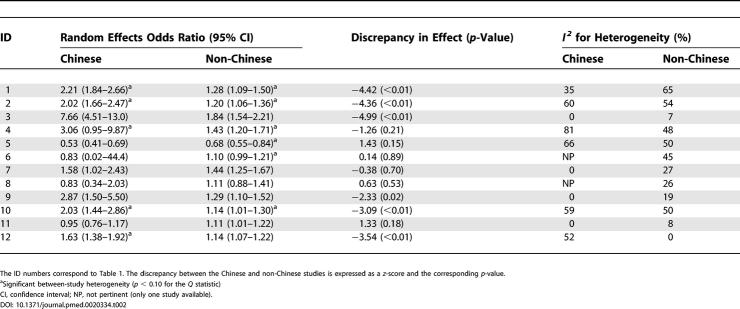
Genetic Effects in Chinese and Non-Chinese Studies

PubMed-indexed Chinese studies were too few for formal comparisons, but the available data suggested that they often tended to provide extreme estimates of genetic effects (Figure 2). In three of the five topics where at least two such studies were available, their summary estimate was the most extreme observed compared with any other group of studies (non-PubMed Chinese, non-Chinese Asian, and non-Chinese non-Asian).

**Figure 2:**
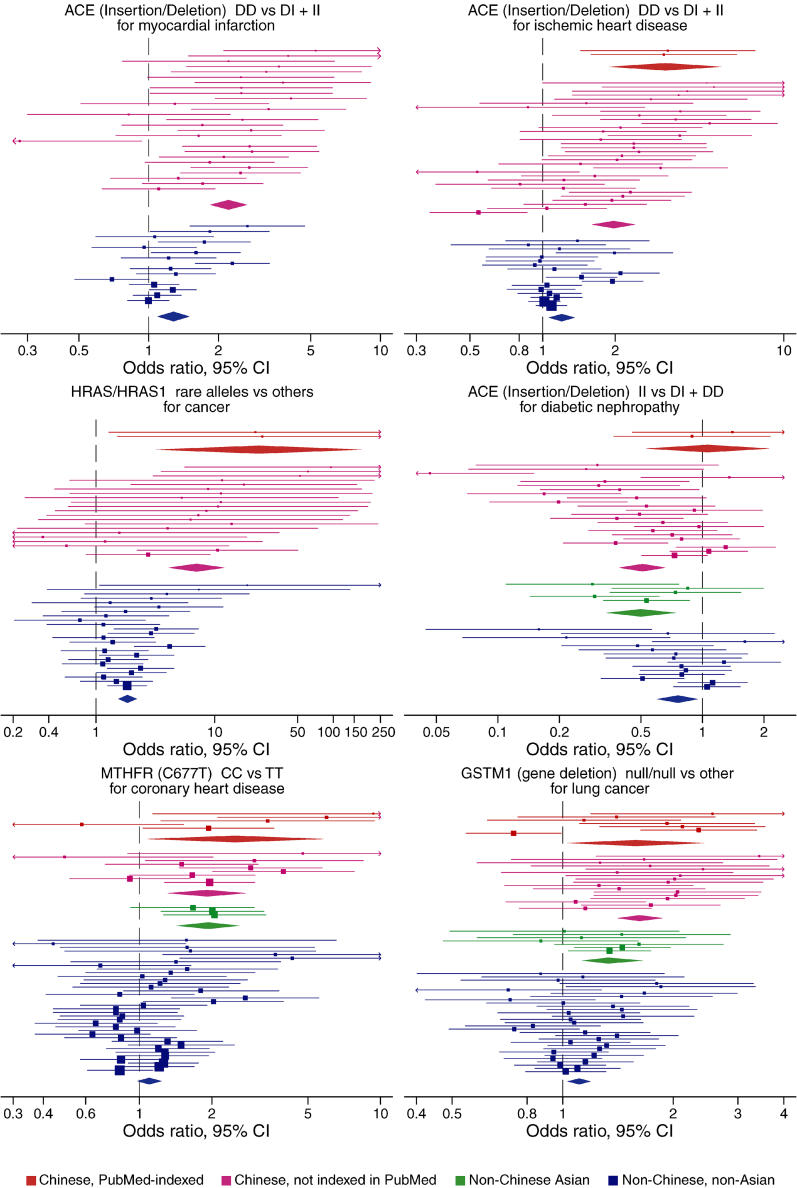
Meta-Analyses of Gene-Disease Associations in a Large Number of Both Non-Chinese and Chinese Studies Each study is shown by its odds ratio and 95% confidence intervals (CIs). The box of the point estimate is proportional to the study weight. Also shown are summary estimates by random effects calculations (diamonds). Summary estimates are obtained separately for Chinese studies indexed in PubMed (red), Chinese studies not indexed in PubMed (pink), non-Chinese studies of Asian descent populations (green), and studies of persons of non-Asian descent (blue). An odds ratio of 1 means no genetic effect, odds ratios larger than 1 mean genetic predisposition, and odds ratios less than 1 mean genetic protection.

Non-Chinese studies of Asian descent populations were available for eight topics. In seven of the eight cases, the estimated genetic effect size was stronger in these Asian-descent studies than in the non-Chinese non-Asian descent studies (Table 3). The difference was beyond chance in two topics (the associations of *MTHFR* C677T polymorphism with coronary heart disease [ID10], and of *GSTM1* gene deletion with lung cancer [ID12]). In topics for which several studies of different groups were available, the non-Chinese studies of Asian-descent populations seemed to have effect sizes somewhere between the effect sizes of Chinese studies and non-Asian studies (see Figure 1).

**Table 3:**
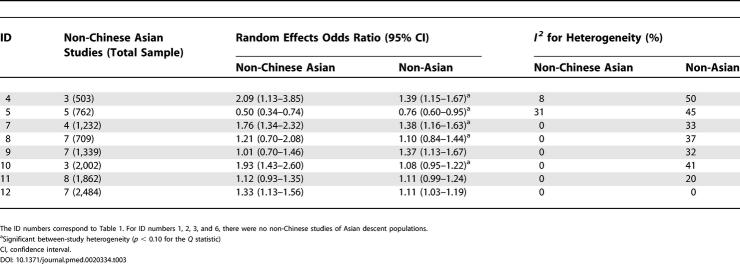
Comparison of Genetic Effects in Non-Chinese Studies of Asian-Descent Populations and Non-Asian-Descent Studies

### Expected versus Observed Significant Findings in Chinese Studies

Power calculations based on asymptotic statistical testing suggested that even if the large summary genetic effects claimed by the Chinese studies were genuine, one would expect 56.6 formally statistically significant studies, substantially fewer than the 78 observed in the database (*p* < 0.001). Based on exact statistical testing, one would expect 61.1 significant studies instead of the 81 observed (*p* = 0.001).

### Qualitative Comparison of Chinese Studies Indexed versus Not Indexed in PubMed

PubMed-indexed Chinese studies did worse than Chinese studies not indexed in PubMed in defining disease with specific criteria (17/20 [85%] versus 137/141 [97%], respectively; exact *p* = 0.042), and in ascertaining the eligibility of controls (13/20 [65%] versus 129/141 [92%], respectively; exact *p* = 0.003). However, the only three Chinese studies mentioning blinding of the genotyping personnel to disease status were PubMed-indexed (exact *p* = 0.002). There was no difference in testing for violations of the Hardy-Weinberg law in the controls (5/20 [20%] versus 38/141 [27%], respectively; exact *p* = 1.00). Only PubMed-indexed Chinese studies had any representation of authors from countries other than China (4/20 [20%] versus 0/141 [0%], respectively; exact *p* < 0.001). As expected, almost all (19/20 [95%]) PubMed-indexed Chinese studies were published in international or national Chinese journals, while only 22 (16%) of the 141 studies not indexed in PubMed were published in national journals.

## Discussion

This empirical evaluation reveals a large Chinese literature on human genome epidemiology that deserves more attention from the international community. The vast majority of this literature does not reach PubMed. Chinese studies usually appear with a time lag of several years after an epidemiological association is first postulated in the world literature, but many such studies are published, especially when the disease is perceived to be common in China. Chinese studies typically suggest much stronger genetic effects than non-Chinese studies, and this may be even more prominent for the few studies that reach PubMed. Although Chinese studies are smaller than non-Chinese studies and thus even more underpowered [5], surprisingly half of them reach formal statistical significance for the evaluated gene-disease association. This exaggeration is seen across very diverse topics.

The larger genetic effects in Chinese studies are unlikely to reflect genuine heterogeneity in the effects of genetic risk factors across various “racial” descent populations [8]. Heterogeneity due to ancestry should not have led always to larger effect sizes in all probed gene-disease associations. Therefore, the most likely explanation is publication bias against “negative” results [29–32] or other selection biases in the chase for statistically significant findings [33]. This explanation is further supported by our analysis of the expected number of statistically significant findings. Even if the average genetic effects in the Chinese studies were indeed as large as those observed, one would expect far fewer Chinese studies to have reached formal statistical significance on their own, given their small sample sizes. The alternative explanation that Chinese investigators may be targeting high-risk populations with particularly strong genetic effects is unlikely given these data.

Language may be a marker for other confounding characteristics of these studies, or even of the whole research and publication milieu. Moreover, even within the English-language studies, strong biases may occasionally operate in the confirmation process. Cultural issues may also be involved with unstated pressures to find positive results for various reasons in different settings around the globe. Various compromises of research quality may ensue.

We focused on gene-disease associations for which a considerable number of studies have been published in the English language. It is possible that there could be a reluctance to submit and publish “negative” or inconclusive results when a large body of English-language literature has shown the presence of genetic effects. Also attempts to confirm multiply supported findings may be more likely to be made, especially with limited resources. However, such pressure for unilateral confirmation destroys the independence and thus also the importance of confirmation.

Our observation is reminiscent also of the randomized trial literature on acupuncture, where studies from China, Russia, Japan, Hong Kong, and Taiwan almost always yielded statistically significant results, in contrast to studies performed in other countries [3]. A predilection for the dissemination of statistically significant results in some non-English speaking countries has also been suspected in other fields, such as lung cancer chemotherapy trials [34]. To our knowledge, there has been no prior documentation of this phenomenon in molecular medicine. Given the rapid pace of production of information in molecular genetics and other modern disciplines, this bias may become a serious problem in the appraisal of cutting-edge science and may jeopardize the credibility of molecular discovery research.

We also found some evidence that superimposed language bias [2] is also operating in this literature. Typically, the few PubMed-indexed Chinese studies showed the most extreme genetic effects, and two-thirds of them reached formally statistically significant results on their own, even though their sample size was very small. Therefore, analyses limited to PubMed-indexed studies may sometimes yield spurious results, if the summary estimates are driven by these extreme findings. PubMed-indexed Chinese studies had worse quality ratings in case and control definitions and ascertainment than Chinese studies not indexed in PubMed. Language bias may not be limited to China, but may also be pertinent to other Asian countries with considerable scientific production, and beyond. We found that non-Chinese Asian studies also tend consistently to show relatively larger genetic effects than non-Asian studies, although data were too sparse to be definitive. The relative extent of selective reporting, publication bias, and language bias is difficult to disentangle here and may vary across topics and across local literatures. It would also be useful to analyze the local literatures for Japanese and Korean studies, where a considerable number of local journals also exist.

The Chinese literature is essential for the evaluation of evidence on genetic risk factors. China is making rapid scientific progress in this field, as in many others. It is already participating in the Human Genome project, and the Southern China National Genome Research Center established in Shanghai in 2001 creates new frontiers for gene-disease association studies. Evidence on population genetics, as well as for any other field pertinent to population health, is extremely important to obtain for China from a global perspective. Moreover, it is unlikely that biases are limited to China, as we discussed above. Also, European and American studies are not necessarily unbiased. There is strong evidence that early-published European and American studies that appeared in the most prestigious journals tended to have inflated results [11,35,36].

Here we did not update further the existing non-Chinese data from the published meta-analyses. Our investigation focused on the Chinese literature, and we tried to focus on meta-analyses with a large number of included studies that should hopefully have reached a stable effect estimate. Nevertheless, for at least two of the postulated associations examined here (the associations of *ACE* with cardiac outcomes), a very large study [37] conducted after the meta-analysis found absolutely no effect, while the previous studies had found modest, but statistically significant, genetic effects. Thus not only was the discrepancy against the Chinese studies even larger than what was found in our analyses, but the evidence from the earlier European-descent studies, in particular the smaller ones [16], had also been biased.

Since most effect sizes in genetic epidemiology, and most other molecular medicine fields, are small or modest, one wonders whether many of the postulated associations are generated from the interplay of various reporting and local literature biases that leave no country immune. In some of these postulated associations, the observed effect sizes may simply be estimates of the prevailing bias [38].

One might argue that the inclusion of poor-quality research may contaminate the better literature rather than provide a more accurate, comprehensive picture. Large-scale aggregate evidence may arrive at erroneous conclusions if studies are automatically included without some critical appraisal. However, it is unfair to judge the quality of research on the basis of its regional origin. Chinese studies may often be as good as or even better than many or most studies from countries publishing routinely in the English language [39–41]. Efforts to improve the quality of research around the globe should run in parallel with enhanced access to global research results.

Our findings have two broad implications. First, language bias may be important to consider in meta-analyses of observational studies in general, and its impact may be as large as or larger than its impact on randomized evidence. Second, human genome epidemiology in particular is a global enterprise, and a critical and comprehensive global view is important to decipher artifacts from true genetic effects. Large studies are useful to validate postulated gene-disease associations [12]. However, such studies are difficult to conduct, they are not completely immune from biases, and their targets must be carefully selected given the plethora of test hypotheses in molecular genetics [5,42]. Besides large studies, registration of investigators and data collections is useful to consider [42,43]. In contrast to randomized trials [44], study registration is impractical in molecular medicine, since investigators would be reluctant to share their hypotheses in public. However, if all investigators working on the genetics of a specific disease were registered in a common network, then it would be easier to trace additional unpublished or non-indexed data. Common networks would also, hopefully, help to improve the quality of research. Such networks should aim for a global, inclusive outlook. The Chinese research output, as well as the output of other non-English-speaking countries, should be appropriately captured. Failure to maintain a global outlook may result in a scientific literature that is driven by the opportunistic dissemination of selected results.

**Patient Summary**

**Background**

There are many different places that medical research can be published. However, some research is never published, which leads to so-called publication bias. One of the biggest divides is between English and non-English research. Research done in non-English-speaking countries can be published in English journals that are usually indexed in major international databases, but more often is published in domestic journals, many of which are not indexed in international databases. This selective publication is called language bias. Scientists have questioned what difference the inclusion or not of non-English studies makes to the total evidence. Publication bias is of concern, especially in genetics, which is a very fast-moving area of research.

**Why Was This Study Done?**

China is a prominent example of a nation with many domestic journals that are not indexed in the international databases. This study looked at Chinese genetic association studies. Understanding the quality and findings of genetics research in China, home to one-fifth of the world's population, is essential for the evaluation of evidence on genetic risk factors. The authors hoped this study would help understand more about selective reporting and language biases.

**What Did the Researchers Do and Find?**

They found that there were many studies (14–35 per topic) for each of the topics they chose to assess, which covered diseases common in China. Generally, Chinese studies appeared a considerable time (two to 21 years) after the first non-Chinese study on the topic. Chinese studies showed stronger genetic effects than non-Chinese studies, despite in some cases having smaller sample sizes. The largest genetic effects were often seen in Chinese studies indexed in Western databases.

**What Do These Findings Mean?**

It seems that there is a combination of selective reporting of studies with interesting findings, and language biases in human gene studies. The main reason most studies didn't appear in the international literature was probably a combination of publication bias and superimposed language bias. It is important to note that such biases are not limited to Chinese literature. Researchers should consider language bias when doing analyses of groups of studies, and efforts to improve the quality of research around the globe should run in parallel with enhanced access to global research results.

**Where Can I Get More Information Online?**

The Cochrane Collaboration has a small section explaining the different types of publication bias:

http://www.cochrane-net.org/openlearning/HTML/mod15-2.htm

BMJ has a presentation on publication bias by a BMJ editor:

http://bmj.bmjjournals.com/talks/publication_bias/index.htm
